# miRNA Profiling: How to Bypass the Current Difficulties in the Diagnosis and Treatment of Sarcomas

**DOI:** 10.1155/2011/460650

**Published:** 2011-02-22

**Authors:** Angélique Gougelet, Jennifer Perez, Daniel Pissaloux, Anthony Besse, Adeline Duc, Anne-Valérie Decouvelaere, Dominique Ranchere-Vince, Jean-Yves Blay, Laurent Alberti

**Affiliations:** ^1^Unité INSERM U590 équipe Cytokines et Cancer, Centre Léon Bérard, 28 rue Laennec, 69373 Lyon cedex 08, France; ^2^Conticanet (FP6-06188), France; ^3^Service d'Anatomie et Cytologie Pathologiques, Centre Léon Bérard, 69373 Lyon cedex 08, France; ^4^EORTC, 83/11 avenue Mounierlaan, 1200 Brussels, Belgium

## Abstract

Sarcomas are divided into a group with specific alterations and a second presenting a complex karyotype, sometimes difficult to diagnose or with few therapeutic options available. We assessed if miRNA profiling by TaqMan low density arrays could predict the response of undifferentiated rhabdomyosarcoma (RMS) and osteosarcoma to treatment. We showed that miRNA signatures in response to a therapeutic agent (chemotherapy or the mTOR inhibitor RAD-001) were cell and drug specific on cell lines and a rat osteosarcoma model. This miRNA signature was related to cell or tumour sensitivity to this treatment and might be not due to chromosomal aberrations, as revealed by a CGH array analysis of rat tumours. Strikingly, miRNA profiling gave promising results for patient rhabdomyosarcoma, discriminating all types of RMS: (Pax+) or undifferentiated alveolar RMS as well as embryonal RMS. As highlighted by these results, miRNA profiling emerges as a potent molecular diagnostic tool for complex karyotype sarcomas.

## 1. Introduction

Sarcomas are rare malignant tumours arising in connective tissues like fat, muscle, bones, and cartilage. According to molecular cytogenetic alterations, sarcomas could be divided into two classes: (1) sarcomas with specific alterations (translocation, oncogenic mutation) including Ewing sarcoma, gastrointestinal stromal tumours, and alveolar rhabdomyosarcoma (2) sarcomas with complex karyotype like leiomyosarcoma, pleomorphic liposarcoma, or osteosarcoma. Osteosarcoma is the most frequent primary malignant bone tumours, characterized by its metastatic potent particularly in lung sites and its resistance to conventional treatments like chemotherapy and radiotherapy [1]. Even if the median survival of osteosarcoma patients has been improved through preoperative administration of chemotherapeutic agents, there are nowadays around 40% poor-responder patients [2]. In fact, osteosarcoma tumours often resist or relapse to presurgical chemotherapeutic treatment, and only few therapeutic options are possible and generally noncurative [3]. A second intensive cure of chemotherapy is currently administered in this case. Thus, it seems essential to develop a diagnosis tool to predict tumour response to chemotherapy to avoid the administration of inefficient drugs. There is also a need for efficient therapeutic alternatives based on the discovery of new targets involved in osteosarcoma tumourigenesis.

Rhabdomyosarcoma (RMS) is one of the most common soft-tissue sarcoma. Three types of RMS are observed: alveolar RMS (20%), embryonal RMS (eRMS, 60%), and pleomorphic RMS (20%). 70% aRMS present a specific translocation of the transcription factor Pax3 at the 3′end of FOXO1, creating a potent transcription factor able to induce myogenesis and survival [4]. 10% aRMS present a translocation of Pax7 with FOXO1 [5]. aRMS are of bad prognosis as compared to eRMS, particularly those with Pax3 fusion gene [6]. Thus, it appears primordial to obtain a diagnosis tool identifying precisely the RMS subtypes, and particularly discriminating Pax-aRMS from eRMS, difficult to separate according to patient survival characteristics, gene expression profiles, and CGH arrays [7].

 Micro-RNAs (miRNAs) are promising diagnosis biomarkers with their tissue specificities and their involvement in oncogenic process [8]. miRNAs are noncoding small RNA molecules synthesized from intronic regions with a size range from 16 to 35 nucleotides. They are processed by specific complexes of proteins containing Drosha and Dicer to be matured and finally integrated in RISC complexes [9, 10]. Mature miRNAs match with complementary sequences in messenger RNAs resulting in translation inhibition and accelerated mRNA degradation [11]. miRNA expression levels are characteristic for one tissue to regulate gene expression during growth and development, as it was shown for skeletal tissue and muscle development [12–14]. Their expression is also deregulated in many cancers [15, 16], resulting in a tumour miRNA signature, which could be useful for their classification in line with their tissue origin and molecular alterations [17–19]. Thus, they currently constitute potent biomarkers for cancer diagnosis [18, 20] with their abilities to be detected in patient serum. A noninvasive diagnostic tool based on miRNAs for osteosarcoma could be very useful to adapt chemotherapy protocols to tumour biological specificities. 

In this study, we performed the miRNA profiling of sarcoma cell lines, human or rat tumours, to assess if miRNAs could constitute potent biomarkers to surpass the current limitations for rhabdomyosarcoma diagnosis and osteosarcoma treatment. miRNA expression levels were determined using microfluidic cards performing high-throughput TaqMan Low Density Arrays (TLDA), a real-time quantitative PCR (RT-qPCR) assays based on TaqMan technology. We firstly studied the effects of different chemotherapeutic agents on osteosarcoma cell miRNA profiles; we observed that these miRNA signatures were cell specific and drug specific. A CGH array of osteosarcoma tumours obtained from a rat model revealed that this miRNA signature, conserved in rat and human cells, was independent of chromosomal rearrangements, suggesting that miRNA profiles were linked to tumour phenotypes rather than to their genetic background. Of great interest, a miRNA signature was identified in rhabdomyosarcoma tumours from patients in accordance with the molecular translocation Pax3 or Pax7. This signature was in fact a potent tool to discriminate alveolar RMS (Pax-) from embryonal RMS, indistinguishable by the molecular techniques currently used. In conclusion, miRNA profiling constitutes a promising technology as an alternative or a partner of usual molecular techniques to overcome the present difficulties in diagnosis and treatment of sarcomas.

## 2. Experimental Procedures

### 2.1. Human Rhabdomyosarcoma Tumours

Seventeen patients treated for rhabdomyosarcoma in the Centre Léon Bérard were included in this study. Four frozen tumours and thirteen formalin-fixed paraffin-embedded tumours were obtained from biopsies realized at the diagnosis. Tumour diagnoses were realized by a referent anatomopathologist specialist for this pathology by immunohistochemistry, FISH, and qPCR.

### 2.2. Cancer Cell Lines

Five cancer cell lines were obtained from ATCC (Manassas, VA, USA): the two human osteosarcoma MNNG/HOS Cl #5 [R-1059-D] (reference CRL-15-47) and Saos-2 (HTB-85) cells, the chondrosarcoma cell line SW1353 (HTB-94) and the two Burkitt lymphoma Daudi (CCL-213) and Namalwa (CRL-1432) cells. Osteosarcoma and chondrosarcoma cells were grown in DMEM (Gibco, Carlsbad, CA, USA), supplemented with 10% decomplemented fetal calf serum (Lonza, Basel, Switzerland), 10 mL penicillin streptomycin (10 U/mL/10 *μ*g/mL, Gibco, Carlsbad, CA, USA), and 5 mL L-glutamin (200 mM; Gibco) at 37°C humidified atmosphere containing 5% CO_2_. Lymphoma cells were grown in RPMI (Gibco, Carlsbad, CA, USA). Cells were exposed to 100 nM RAD-001 (Novartis), 50 *μ*M ifosfamide (ifos, Baxter) or 1 *μ*M cisplatin (CDDP, TEVA) or 100 *μ*M methotrexate (MTX, TEVA) for 24, 48, and 72 h.

### 2.3. Rat Osteosarcoma Model

Procedures for animal care were performed according to institutional and national guidelines. Animals were anesthetized throughout all surgical and imaging procedures with isoflurane/oxygen (2.5%/2.5%, v/v) (Minerve, Esternay, France). The transplantable orthotopic and metastatic rat osteosarcoma model has been previously described [21–23]. This model mimics its human counterpart in terms of aggressiveness, metastatic spreading and chemoresistance phenotype [21–23]. All the tumours obtained were classified as osteoblastic following histological analyses. Briefly, small tumour fragments (100 mm^3^) taken from a hyperproliferative osteogenic tumour area were grafted on 3-weeks old immunocompetents Sprague-Dawley rats (Charles River Laboratories, Wilmington, MA, USA). Using a lateral approach, a tumour fragment was placed contiguous to tibial diaphysis after periosteal abrasion; then, the cutaneous and muscular wounds were sutured. Fourteen days after tumour transplantation, animals underwent a first ^18^F^−^FDG PET Scan and were randomly assigned to a control group treated with saline solution or a treated group exposed to a subcutaneous dose of 10 mg/kg ifosfamide (ifos, Baxter, Deerfield, IL, USA), 7 days apart (at days 15 and 22 after tumour transplantation). A second ^18^F^−^FDG PET Scan was performed 7 days after the second ifos administration. Animals were sacrificed one week after the end of the treatment. Tumour and normal tissue fragments (muscle, bone, and lung) were collected for RNA extractions.

### 2.4. RNA Extraction and Quantitative Real-Time PCR

FFPE tumours were lysed for 24 h in ATL buffer (Qiagen, France) supplemented with proteinase K (Qiagen) at 60°C in rotative agitation after different washes with toluene, ethanol, and tris/EDTA in this order. Total RNA was extracted from tumour or cell pellets using a single phenol/chloroform extraction protocol with Trizol, according to the manufacturer's instructions (Invitrogen, Carlsbad, CA, USA). Five hundred nanograms of total RNA were subjected to the microfluidic PCR technology performed by Applied Biosystems (Foster City, CA, USA). In brief, RNA was reversed transcribed, using multiplexed specific looped miRNA primers from the Taqman MicroRNA Reverse Transcription kit. The second step consists in a real-time quantitative PCR on TLDA: RT products are introduced through microchannels into miniature wells that are preloaded with dehydrated specific primers and probes. Recently, Applied biosystems released the second version of TLDA, consisting of two cards A and B. Analyses were performed for 377 miRNAs on card A and 290 on card B.

### 2.5. PCR Data Normalization

For each miRNA, the threshold cycle (Ct) was calculated by the ABI 7900 Sequence Detection System software (plate by plate manual Ct analysis with a threshold at 0.25 and automatic baseline). All further data manipulations were done using R scripts. A cutoff of 32 was applied to discard the late Ct values, except for RMS analysis. Around 60% of miRNAs passed the filtering criteria and were used for further analysis. For each TLDA, quality controls were performed on the raw data by checking internal controls and using box plot and scatter plot diagrams. Samples with any kind of problems were discarded so they would not introduce bias during the following normalization procedures. We tested different methods of normalization since the recommended “pseudo” normalization factor mammU6 plotted in each card was not stably expressed in our different samples. Normalization with the two most stable miRNAs identified by GeNorm was not optimal too. Finally, a global normalization by the median was chosen for its reliability over experiments. Tissues included in a given analysis were treated altogether, the normalization procedure being applied separately for the two types of card, A and B. Distribution of normalized data was checked with box plots and correlation plots. The following formula was used to correct Ct values of every card:

(1) $$\left(\text{Normalized Ct}\right)=\frac{\text{Ct}\times \left(\text{mean of medians}\right)}{\left(\text{median of the card}\right)}.$$

Through this approach, the new median value shared by all samples can be considered as a sort of perfect “virtual housekeeping gene”. Therefore, the standard ΔΔCt method can be used to determine the relative quantities (RQ) as follows:

(2) ΔCt  =  (Normalized Ct)−(New shared median).

For the ΔΔCt calculation, it was more relevant for the statistical analyses to use the mean of all ΔCt obtained across samples for each miRNA, instead of using the ΔCt of a reference sample

(3) $$\begin{array}{cc} & \Delta \Delta \text{Ct}\\ & =\Delta \text{Ct}\;-\left(\text{Mean of }\;\Delta \text{Ct across samples for each miRNA}\right), \end{array}$$

(4) $$\text{RQ}=\;2^{-(\Delta \Delta \text{Ct})}$$

### 2.6. miRNA Target Predictions

We compiled 4 databases to determine miRNA targets: TargetScan 5.1, MiRanda, PICTAR, and the miRbase databases. These databases search the presence of conserved 8mer and 7mer sites on the 3′UTR parts of messenger RNA that match the seed region of each miRNA. It also predicts the efficacy of targeting for each matching site. We created our own database which regrouped each miRNA with the geneID of all their protein targets, for rat and human. We only conserved couples miRNA/geneID present in two databases at least.

### 2.7. miRNA-Regulated Cell Signalling Pathways Predictions

We used the “G-language microarray” web application, which allows the mapping of molecular dataset onto “Kyoto Encyclopedia of Genes and Genomes” (KEGG) pathway maps [24]. We first input miRNA-targeted proteins of interest and the sum of RQ values for all miRNAs that regulate these proteins, contained between 1 and 50; the software then generates KEGG data to create FLASH graphics of cell signalling pathways in which proteins are involved. The colour intensity of a highlighted protein varies with the strength of its regulation by miRNAs.

### 2.8. Proliferation Assay

Cells were plated in 96 well plates at 5000 cells/well and exposed to 100 nM RAD-001, 50 *μ*M ifosfamide, 100 *μ*M methotrexate, or 1 *μ*M cisplatin or not (NT). Cell growth was measured 24, 48, and 72 h later with 20 *μ*L Cell Titer Glo luminescent reagent (Promega, Madison, WI, USA) for 10 min. Luminescence was recorded using a Microbeta reader (PerkinElmer, Fremont, CA, USA).

### 2.9. Western Blot

Pelleted cells were resuspended in lysis buffer (Tris 50 mM pH 7.4, NaCl 250 mM, EDTA 5 mM, NaF 50 mM, Triton X-100 0.1%, orthovanadate 1 *μ*M) plus protease inhibitors for 30 min on ice. After a centrifugation at 14000 rpm for 10 min, supernatants were boiled for 5 min in Laemmli sample buffer (Biorad, Hercules, CA, USA). Analysis of protein content was performed on 4%–12% gradient gel. After electrophoretic separation, 30 *μ*g proteins were electrotransferred on a polyvinylidene difluoride membrane (Immobilon P, Millipore corp., Bedford, MA, USA). The membrane was then blocked for 1 h at room temperature with blocking agent 0.2% in PBS/Tween 0.1%, probed overnight with a primary rabbit antibody against the protein of interest, and finally revealed with a secondary antirabbit antibody HRP conjugated (Upstate Biotechnology, Lake Placid, NY, USA) and ECL Advance system (GEhealthcare, Chicago, IL, USA). Primary antibody used was obtained from Cell Signaling (New England Biolabs, Beverly, MA, USA) used at 1/1000. The *β* actin was used as a reference (Sigma).

### 2.10. CGH Array

Oligonucleotide-based microarray analysis was performed using a custom-designed, 244K-feature whole-rat genome microarray manufactured by Agilent Technologies (Santa Clara, CA). Genomic DNA labeling, array hybridization, and washing were performed as specified by the manufacturer (Agilent Technologies). Results of aberration calls consisting of three or more consecutive oligos were then displayed using custom oligonucleotide CGH analysis software (Genespring).

### 2.11. Statistical Analysis

Normalized RQ data were directly input into the TIBCO Spotfire DecisionSite for Functional Genomics analysis software. We performed unsupervised hierarchical clustering to classify samples by groups. The selection of miRNAs useful to predict tumour response to treatment was statistically realized using ANOVA tests with *P* values of  .05 at least. Results were verified through supervised hierarchical clustering.

 Data from miRNA lists of interest were then used as variables in a three-dimensional principal component analysis (PCA) performed with R 2.9.0 package to demonstrate their capabilities to distinguish types of tumours. PCA supplies a simplified three-dimensional picture to our multivariate dataset of miRNA RQ values. By mathematical combination of values according to their strength, three principal components are created that represent as much as possible the variability of the data. Thus, tumours possess three new coordinates in a three-dimensional space. According to their localization in this space, tumours form groups, and their subtypes can be predicted.

## 3. Results

### 3.1. miRNA Signatures of Osteosarcoma Cell Lines

In our recent study published in International Journal of Cancer, we showed that the two osteosarcoma Saos-2 and CRL-15-47 (15-47) cells mimic the biological response of human osteosarcoma and tumours obtained from a rat model. In fact, we identified in an osteosarcoma rat model a panel of 61 miRNAs discriminating tumours with a good response to ifosfamide from those with a bad response [25]. On the basis of this signature, we realized a principal component analysis allowing predicting tumour response. In this PCA diagram, we could notice that the Saos-2 cells were predicted as sensitive to ifosfamide contrary to 15-47 cells (Figure 3(b) [25]), according the results obtained by a proliferation assay (Figure 6(a) [25] and Figure S1). This was confirmed by a PCA analysis realized with the miRNA signature identified in human tumours (Figure S2). We so considered that these two cell lines were an interesting model to study the importance of miRNAs in cell response to treatment and to identify new therapeutic strategies.

### 3.2. miRNA Signatures of Human Cancer Cell Lines

**Figure 1:**
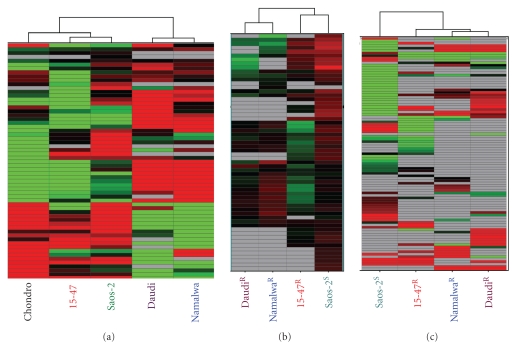
Cancer cell miRNA signatures were consistent with their tissue origin and with their sensitivity to ifosfamide. (a) This unsupervised hierarchical clustering only conserved the 61 miRNAs which discrimated osteosarcoma cells according to their response to treatment. Osteosarcoma cell lines clustered together near chondrosarcoma cells and independently to the lymphoma Daudi and Namalwa cells. Each row represents the relative levels of expression for each miRNA, and each column shows the expression levels for each sample. The red or green colour indicates relatively high or low expression, respectively, while grey squares indicate no expressed miRNA. (b) and (c) miRNA profiles after exposure to 50 *μ*M ifosfamide for 24 h. (b) This unsupervised hierarchical clustering only conserved the 61 miRNA differently expressed in osteosarcoma cells according to their sensitivity to ifos following an ANOVA (*P* < .03). (c) This supervised hierarchical clustering conserved miRNAs differently expressed in cells according to their response to treatment following an ANOVA (*P* < .05) after removing the miRNAs whose expression depends on the cell types. The red or green colour indicates relatively high or low expression, respectively, while grey squares indicate no expressed miRNA.

We firstly performed a preliminary miRNA profiling on different cell models to compare the miRNA profiles of osteosarcoma cells used in our laboratory to perform *in vitro *experiments, Saos-2 and 15-47 cells, with the chondrosarcoma cells SW-1353 (chondro) and the Burkitt lymphoma Daudi and Namalwa cells. In a previous study, we identified 61 miRNAs involved in osteosarcoma cell response to treatment [25]. We only conserved these miRNAs to realize an unsupervised hierarchical clustering with the five cancer cell lines. As shown in Figure 1(a), this miRNA signature was representative of the two human osteosarcoma cell lines, since these two cells clustered together independently but closely to the chondrosarcoma cells. These three cell lines were classed in a distinct group from the two lymphoma cells Daudi and Namalwa. This confirmed that each cancer cell line presents a miRNA signature in accordance with their origin, as shown by others [15, 16].

### 3.3. miRNA Profiles in Response to Chemotherapeutic Agents Were Cell Specific

Then, we assessed if miRNA profiles were specifically modified in response to chemotherapy. We chose to expose osteosarcoma and lymphoma cells to ifosfamide, an alkylating chemotherapeutic agent currently used for paediatric osteosarcoma. A proliferation assay based on ATP measurement showed that the only Saos-2 cell line was moderately sensitive to 50 *μ*M ifosfamide after 48 h exposure (proliferation inhibition around 30%) (Figure S1). Based on this observation, we decided to expose these cells to 50 *μ*M ifosfamide for 24 h to realize miRNA profiling. On the basis of the panel of 61 miRNAs identified in our previous study [25], osteosarcoma cells were markedly different from lymphoma cells, confirming that miRNA profiles were cell specific as shown by the unsupervised hierarchical clustering in Figure 1(b). We could notice that Saos-2 cells present a unique miRNA signature in which the majority of miRNAs were overexpressed (in red in Figure 1(b)). A supervised hierarchical clustering realized following an ANOVA *P* < .03 between the Saos-2 sensitive cells versus the resistant cells revealed that they effectively clustered according to their sensitivity to ifos: Saos-2 in one hand, independently to 15-47 cells and both lymphoma cells (Figure 1(c)). We confirmed this observation with the other chemotherapeutic agent ciplatin. As previously, cells were classified according to their susceptibility to CDDP on the supervised hierarchical clustering in Figure S3A (ANOVA *P* < .03): the 15-47 and Namalwa cells, sensitive to CDDP based on the proliferation assay in Figure S3B, clustered together, independently to Daudi and Saos-2 cells refractory to this treatment.

### 3.4. Osteosarcoma Cell miRNA Profiles Were Specific of Each Chemotherapeutic Agent

**Figure 2:**
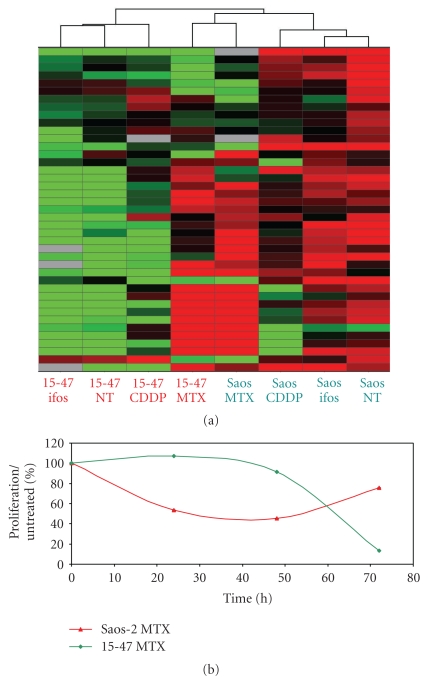
miRNA expression profiles osteosarcoma cells were specific for each chemotherapeutic agent. (a) This unsupervised hierarchical clustering conserved the miRNAs identified as discriminating for ifosfamide response after removing the miRNAs whose expression depends on the cell types. Each row represents the relative levels of expression for each miRNA, and each column shows the expression levels for each sample. The red or green colour indicates relatively high or low expression, respectively, while grey squares indicate no expressed miRNA. (b) Cell growth was measured by the Cell Titer GloLuminescent assay as described in Section 2.6 24, 48, and 72 h after exposure to 100 *μ*M methotrexate. Results were represented as the mean % of proliferation normalized to untreated cells of two independent experiments realized in duplicate.

Thus, since miRNA signatures of untreated as well as treated cells were cancer specific, we assessed if each chemotherapeutic drug induced a different miRNA profile in a same cell. As suggested previously for osteosarcoma cells, cisplatin and ifosfamide exposure resulted in quite different miRNA profiles. After a statistical analysis with an ANOVA *P* < .03, we only found two dicriminating miRNAs common to both miRNA signatures induced by ifos and CDDP in the two cell lines (Figure S3). In this context, we test a third cytotoxic agent currently administered in osteosarcoma pathology, the methotrexate. As shown in the unsupervised hierarchical clustering in Figure 2, only conserving the 61 miRNAs of interest for osteosarcoma response, as explained above, the miRNA signature in the two osteosarcoma cells Saos-2 and 15-47 strongly differed from those observed for ifsofamide and cisplatin. It is important to note that a majority of these miRNAs were overexpressed in both cell lines in response to MTX. This was relevant with their sensitivity to MTX as shown in the proliferation assay in Figure 2(b). In brief, it seems that discriminating miRNAs were generally overexpressed in the cells after exposure to a cytotoxic agent, to which they were sensitive, as it was also shown for ifosfamide in the Saos-2 cells (Figure 1(b)). This also confirmed that miRNAs predicting cell response to a treatment differed according to the drug. 

On the basis of these preliminary *in vitro* results, we could suggest that miRNA profiles, due to their drug specificity, could be a potent tool to predict a cancer cell response to a treatment. Since osteosarcoma is currently resistant to conventional treatments, the prediction of its response to one agent could be a progress for this pathology.

### 3.5. Osteo- and Chondrosarcoma Cell Response to the mTOR Inhibitor RAD-001

As highlighted by these previous data, we were able to classify and predict osteosarcoma cell response to chemotherapy. Our algorithms were not only interesting for chemotherapeutic agents but also promising to identify new targeted therapies to encounter osteosarcoma resistance. Thus, we tested a potent drug for skeletal sarcoma treatment, which inhibits the pro-oncogenic protein mTOR, called RAD-001 (Everolimus, Novartis). mTOR is often aberrantly activated in cancers and, in particular in chondrosarcoma [26] and osteosarcoma [27]. mTOR signalling has been described as implicated in tumour development, metastasis, and drug resistance [28, 29]; thus, mTOR targeting successfully inhibits tumour growth and renders them sensitive to conventional treatments [30, 31]. RAD-001, acting in a similar manner than rapamycin through the inhibition of mTORC1 complexes, is currently tested in various clinical trials for renal cell carcinoma (RECORD program), advanced papillary tumours (RAPTOR), metastatic neuroendocrine tumours (RAMSETE), or breast cancers (BOLERO). 

**Figure 3:**
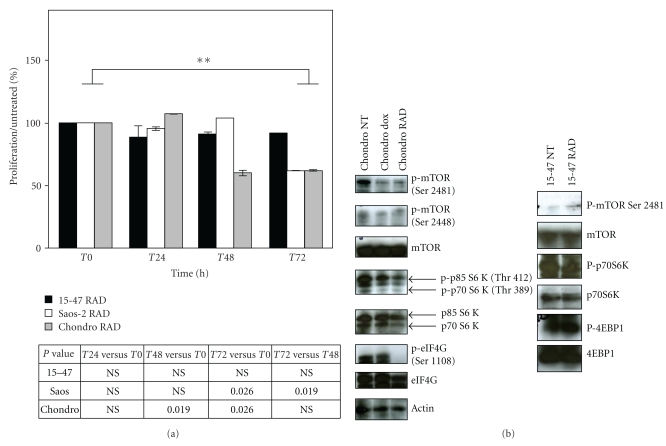
mTOR inhibition by RAD-001 in chondrosarcoma resulted in cell proliferation inhibition. (a) Cell growth was measured by the Cell Titer GloLuminescent assay as described in Section 2.6 24, 48, and 72 h after exposure to 100 nM RAD-001. Results were represented as the mean % of proliferation normalized to untreated cells of two independent experiments realized in duplicate. A Fisher test was realized, NS corresponds to “nonsignificant”. (b) Western blot analysis of RAD-001 effects on the mTOR pathway in the chondrosarcoma and 15-47 osteosarcoma cells exposed or not (NT) to doxorubicin (dox). 30 *μ*g protein extracts were analysed by Western blot with antibodies 1/1000 against actors of the mTOR pathway (phosphorylated form or not) (Cell signalling, Beverly MA).

Thus, we performed *in vitro *experiments on chondrosarcoma and osteosarcoma cells with 100 nM RAD-001. The Saos-2 and chondrosarcoma cell proliferation was reduced of 40% following exposure to RAD-001 during 72 h contrary to 15-47 cell growth (Figure 3(a)). In parallel, we realized Western blot with RAD-001 on chondrosarcoma and osteosarcoma cells concerning the major actors of the mTOR cell signalling pathways. This revealed that the mTOR pathway was inhibited by RAD-001 in chondrosarcoma cells contrary to 15-47 cells, in particular eIF4G and p70 S6 kinase whose phosphorylation level was decreased (Figure 3(b)).

**Figure 4:**
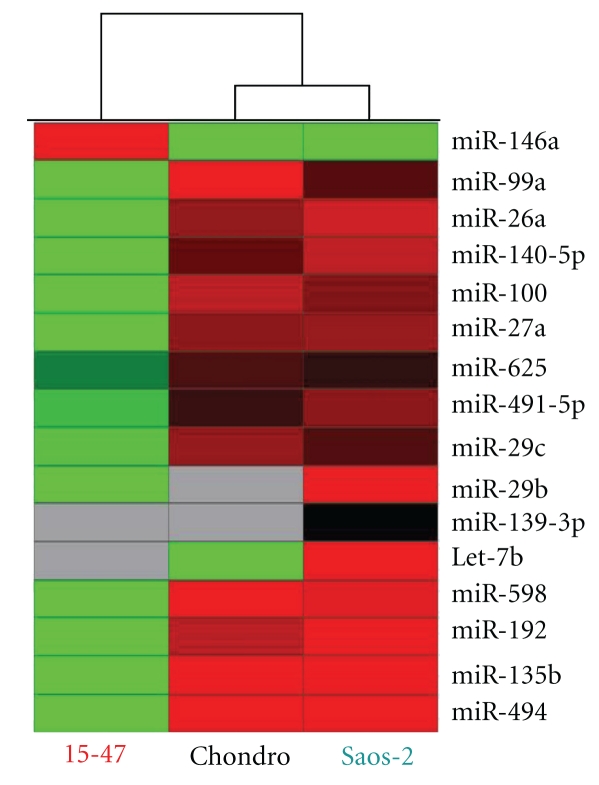
miRNA expression profiles of osteosarcoma and chondrosarcoma cells were consistent with their sensitivity to the mTOR inhibitor RAD-001. This hierarchical clustering only conserved miRNA differently expressed in tumours according to their response to treatment following an ANOVA (*P* < .05). Each row represents the relative levels of expression for each miRNA and each column shows the expression levels for each sample. The red or green colour indicates relatively high or low expression, respectively, while grey squares indicate no expressed miRNA.

Thus, we analysed if the miRNA signatures of these cells were different and could explain their differential response to RAD-001. We performed a supervised hierarchical clustering between untreated Saos-2, chondrosarcoma and 15-47 cells following an ANOVA with *P* < .05. This clustering revealed that 16 miRNAs discriminated the chondrosarcoma and Saos-2 cells in one hand and the 15-47 cells in the other hand (Figure 4). Except miR-146, Saos-2 and chondrosarcoma overexpressed these contributory miRNAs. 

**Figure 5:**
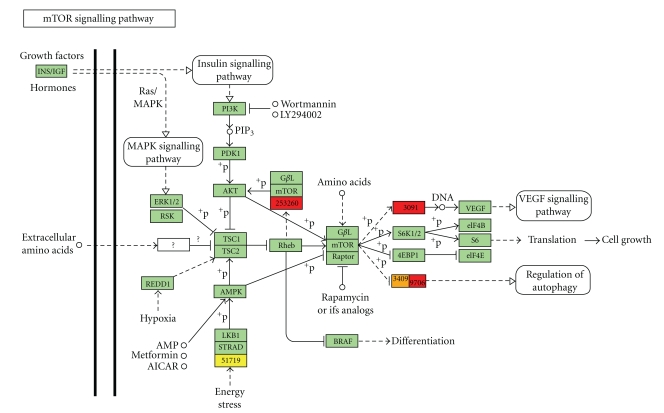
The discriminating miRNAs interfered with the mTOR pathway. Proteins in yellow, orange, and red colours represent targets of miRNAs; a yellow square represents a weak repression, while red represents the maximal repression; green squares were not targeted.

Thereafter, as we have explained in our previous study on osteosarcoma [25], miRNA profiling constitutes a potent tool to identify miRNA-targeted cell signaling pathways through an *in silico *approach. In our case, we searched if these miRNAs shown as differently expressed in cells according to their response to RAD-001 potentially target the mTOR signalling pathway. We created a database, as described in Section 2.6 which determine the predicted targets for these miRNAs described in the miRbase. Then, we summed up the RQ values for each miRNA in the Saos-2 and chondrosarcoma cells sensitive to RAD-001 and concatenated with the geneID of their protein targets. We finally inserted these data in the G-language microarray web application, which connects miRNA targets according to their involvement in similar KEGG pathways, the mTOR pathway in this case. As shown in Figure 5, the mTOR pathway is targeted by these miRNAs and particularly its downstream proteins implicated in VEGF signaling and autophagy processes, in particular RICTOR, ATG1, and HIF-1a. Thus, the Saos-2 and chondrosarcoma cells overexpressed miRNAs that potentially inhibit mTOR signalling. Inhibition of these miRNAs through the use of Locked Nucleic Acid (LNA) and qPCR measurement of RICTOR, ATG1, and HIF1a could confirm this concept. 

To resume, miRNAs constitute potent biomarkers to determine the susceptibility to a treatment and could be very useful to identify new therapeutic targets as an alternative of chemotherapy for chondrosarcoma and osteosarcoma often refractory to this treatment. In the next steps, we assessed if these observations were relevant *in vivo*, with a model of rat osteosarcoma and with patient samples.

### 3.6. Predictive miRNA Signature of a Rat Osteosarcoma Model Was Probably Not Related to DNA Aberrations

**Figure 6:**
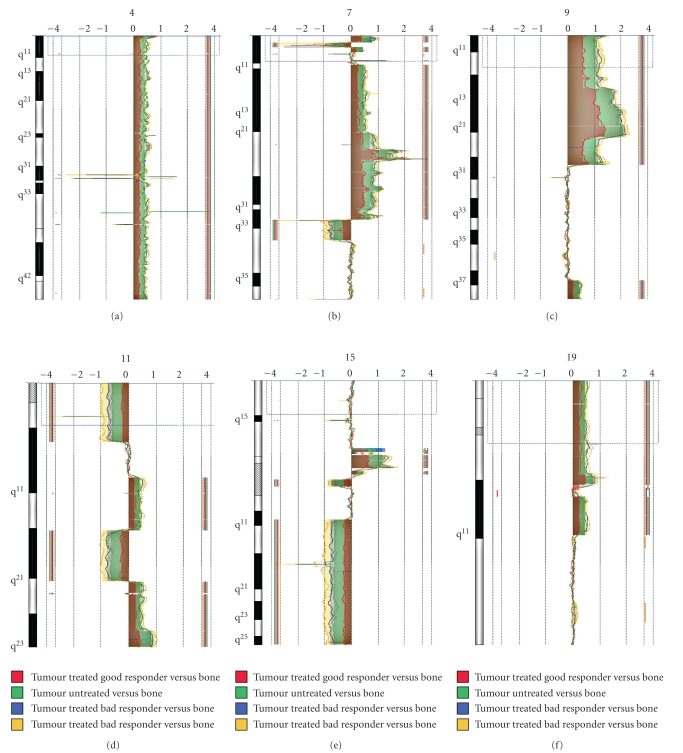
CGH analysis of six tumours from an osteosarcoma rat model. Here, we represent six chromosomes among the twenty + X chromosomes present in rat genome. All the analyses were performed with the same untreated bone sample as a reference.

As described in other studies realized by members of our team [21, 22, 25], we possess a rat osteosarcoma model mimicking the human pathology concerning aggressiveness, chemoresistance and the apparition of lung metastases (see Section 2.6). The treatment of animals with ifosfamide results in two groups, the good versus the bad or moderate responders, in a proportion closer to that observed for patients. By miRNA profiling, we were able to distinguish tumours sensitive to ifosfamide from those refractory to this drug and above all to predict the response of untreated tumours with ten miRNAs through the use of statistical algorithms created in our lab [25]. Following these interesting data, we would like to confirm that this miRNA signature was specific of tumour response to treatment and not related to different tumour genetic backgrounds. We thus realized an analysis in CGH array with the same tumours used for miRNA profiling. We analysed two tumours of each type, untreated, treated with ifosfamide and good responder, or treated with ifosfamide and bad responder, as compared to the same untreated bone sample, the reference tissue in CGH analysis. The majority of chromosomal aberrations observed in CGH array was common to untreated tumours and treated tumours, regardless of their response to treatment (Figure 6). The few different abnormalities were essentially linked to individual tumour biological specificities. 

We compiled all abnormalities and verified in our “home-made” database if any miRNA, identified as discriminating of tumour response, was located in these DNA regions. Interestingly, this *in silico *analysis also revealed that neither miRNA nor gene were present in the few differential aberrations observed in these tumours, in particular in the chromosome 4 (Figure S4), suggesting that the different miRNA profiles were rather linked to tumour response to treatment and not due to upstream chromosomal rearrangements. Although we could not rule out that some trans-acting proteins could be deregulated consequently to these aberrations, this suggested that all tumours were homogeneous and that an increase in some miRNAs in sensitive tumours were due to their upregulation and not to a genetic amplification. 

It seems that molecular diagnosis based on miRNA profiling highlights the tumour behaviour, that is, in response to a treatment, and thus a phenotype rather than a genotype contrary to CGH array. These two molecular techniques could be a couple of choice to improve the care of patients with pathologies currently hardly to diagnose.

### 3.7. Rhabdomyosarcoma miRNA Profiles Were Correlated to their Histological Subtypes

**Figure 7:**
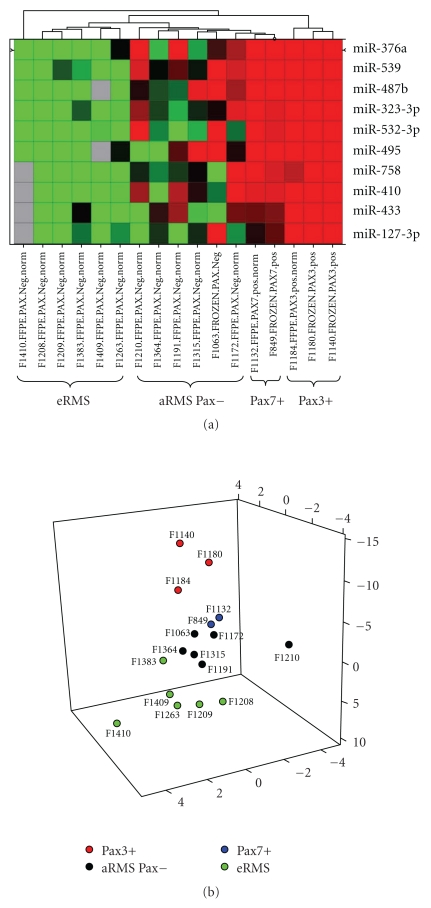
Rhabdomyosarcoma miRNA signatures were consistent with their molecular alterations. (a) This supervised hierarchical clustering only conserved miRNA differently expressed in the different subtypes of RMS following an ANOVA (*P* < .03). Each row represents the relative levels of expression for each miRNA, and each column shows the expression levels for each sample. The red or green colour indicates relatively high or low expression, respectively, while grey squares indicate no expressed miRNA. (b) Principal component analysis of RMS tumours as a tool to determine the potential tumour response to treatment. RQ values of the ten selected miRNAs for each tumour were corrected by the coefficients determined in (a), as described in Section 2.6.

Finally, to corroborate the previous idea considering that miRNA profiling could be very helpful for uncertain diagnoses, we performed the miRNA profiling of rhabdomyosarcoma samples. In fact, we recently showed that miRNA profiling was reliable for osteosarcoma diagnosis on 29 formalin-fixed paraffin embedded (FFPE) biopsies of patients [25]. Based on the expression level of a panel of five miRNAs, we successfully separated good responders from bad responders to treatment. So, we assessed if our TLDA platform was also competitive for RMS diagnosis. We obtained seventeen tumours including alveolar RMS patients, (Pax3+) (3 patients) or Pax7+ (2), embryonal RMS patients (6) and negative fusion aRMS (6). All these tumours were diagnosed through the use of immunohistochemistry, FISH and qPCR, which were validated by a referent anatomopathologist (Table S5). A supervised hierarchical clustering on rhabdomyosarcoma tumours following an ANOVA with a *P* value <.03 between the four types of RMS, revealed that tumours clustered according to their molecular alterations Pax3/FOXO1, Pax7/ FOXO1 or no translocation, on the basis of the expression level of 10 miRNAs (Figure 7(a)). (Pax+) tumours, particularly those (Pax3+) overexpressed all these miRNAs. Then, we performed a statistical analysis with these ten miRNAs based on Principal Component Analysis, a method which allows studying the variability between a set of variables. This consists of assigning a new system of three coordinates to each contributory miRNA by a mathematical procedure. Then, RQ values of each miRNA are adjusted for each tumour by the new coefficients obtained previously and summed up. Thus, a 3-dimension PCA diagram was realized with the three new coordinates for each tumour (Figure 7(b)). Through this mathematical representation, we could distinguish (Pax+) from fusion negative aRMS and eRMS. eRMS also constitutes an independent group with a high value of component 2 (represented in the y-axis on Figure 7(b)). The fusion negative aRMS constitute a separate group even if some samples were difficult to classify in accordance with their uncertain diagnosis. Even if the number of samples was low for each subset, a statistical analysis showed a significant *P* value between (Pax3+) and (Pax7+) and between (Pax+) and (Pax−) tumours, 0.05 and 0.0005 respectively (Figure S6).

We showed that miRNA profiling was a potent tool to discriminate fusion negative aRMS from embryonal RMS. miRNAs could be useful biomarkers to improve the diagnosis of this type of RMS, since fusion negative aRMS are currently molecularly indistinguishable from eRMS [7].

## 4. Discussion

miRNA signatures are observed for many types of cancers, that is, sarcoma [19], breast and prostate cancers [18, 32]. These signatures constitute potent diagnosis and prognosis tools for chronic lymphocytic leukemia [33], colon adenocarcinoma [34], or lung cancers [35]. Here, we showed that osteosarcoma cell lines also expressed miRNA patterns different from those of chondrosarcoma and lymphoma cells (Figure 1(a)) and which allow us to discriminate cell response to chemotherapeutic treatment (Figures 1(b), 2, and S3). In addition, osteosarcoma miRNA signatures were cell and drug specific (Figure 2(a)). This drug specificity of osteosarcoma has also been observed by Song et al. with U2-OS osteosarcoma tumour xenografts, in which different miRNAs were deregulated in response to the chemotherapeutic agents doxorubicin, cisplatin, and ifosfamide; only 3 miRNAs were commonly found deregulated in response to all drugs [36]. With their specificity, miRNAs constitute promising biomarkers to anticipate the tumour response to a treatment of interest. As we have recently shown, through miRNA profiling, we were able to predict osteosarcoma tumour response to chemotherapy for rat tumours as well as for patient FFPE biopsies [25]. Here, we showed that miRNA profiles of osteosarcoma cells were in accordance with their response to the mTOR inhibitor, RAD-001 (Figures 3 and 4). The miRNAs deregulated in response to this drug in sensitive cells, effectively targeted the mTOR pathway, in particular the downstream proteins eIF4G and p70 S6 kinase (Figure 3(b)), and potentially RICTOR, ATG1 and HIF1a, which might be validated by qPCR analysis (Figure 5).

 In brief, miRNAs appeared very useful for the identification of new exciting therapeutic approaches through the targeting of some miRNA protein targets or some miRNAs involved in tumour development themselves. In future, we would like to confirm the implication of these miRNAs in treatment response *in vitro* through the use of miRNA mimics or inversely of Locked Nucleic Acid (LNA) against these miRNAs. As mentioned in this study, we possess an interesting *in vitro* osteosarcoma model, on which we could test the miRNA functionality in the presence of the different drugs used in this work. Following the validation of miRNA involvement *in vitro*, we would also test these mimics or LNAs *in vivo* in the model of rat osteosarcoma. This approach has been successfully employed in rhabdomyosarcoma through the conditional expression of miR-206 in mice [37] and could become a potent therapeutic strategies [38].

In addition to the identification of new targets, miRNA also constitute an interesting alternative to the conventional molecular technologies routinely used for cancer diagnosis. In fact, osteosarcoma present complex karyotypic alterations rendered them difficult to diagnose with current diagnostic methods, like CGH array [39]. With the rat osteosarcoma model, we confirmed that tumours presented numerous long chromosomal aberrations (Figure 6). These abnormalities were generally common to all tumours, regardless to their susceptibility to treatment and neither miRNAs of interest nor genes were located in these regions (Figure S4). Even if some proteins involved in the regulation of miRNA expression (trans-acting factors or epigenetic regulating factors) could be deregulated following these mutations, the miRNA profiles observed in rat tumours might be correlated to the effects of the cytotoxic drugs on the miRNA machinery and no to upstream DNA rearrangements. Even if miRNAs could be submitted to epigenetic regulation like methylation or acetylation, this only concerns 5% to 10% miRNAs, and we could consider that this process is minor for the miRNA signature of osteosarcoma tumours and cells based on 61miRNAs [40, 41]. Enthusiastically, our work is the first suggesting that miRNA signatures were not correlated to DNA amplifications, as it was observed for neuroblastoma [42] or in mixed lineage leukemia [43]. Although our cohort was not fully satisfying, it appeared that miRNA profiling could predict tumour response to treatment by reflecting tumour biological specificities and not genotypic characteristics. This work also highlights miRNA measurement as an interesting partner to CGH array in the case of pathologies with unstable karyotypes. In the same way, Selvarajah et al. was the first to suggest a combination of CGH array and interphase FISH to better understanding osteosarcoma pathogenesis [44]. 

miRNA patterns were not only related with osteosarcoma phenotypic properties but also with rhabdomyosarcoma histological subtypes. By miRNA profiling, we were able to discriminate the different subtypes of rhabdomyosarcoma: Pax3+ or Pax7+ or fusion negative, classically difficult to diagnose by histological analysis (Figure 7(a)). This miRNA pattern was unique since all miRNAS identified as discriminating are no or weakly described in the literature. Very interestingly, on the basis of their miRNA profiles, our algorithms allow us to discriminate embryonal RMS from fusion negative aRMS (Figure 7(b)). It was in agreement with the work of Wachtel et al. identifying different expression profiles linked to aRMS (Pax+), fusion negative aRMS, and eRMS [45].

Altogether, it seems that miRNA measurement is advantageous for sarcoma with complex karyotype, since fusion negative RMS, similarly to osteosarcoma, are characterized by a complex karyotype linked to allelic imbalance, loss of heterozygoty and heterogeneous gene expression profiles. Although the molecular classification of fusion negative RMS is always controversial, our work corroborates the study of Davicioni et al. suggesting that Pax/FOXO1 dictates a specific expression signature in RMS by oligonucleotide microarray expression profiling [46]. Inversely, this differs from the recent work of Williamson suggesting that fusion negative alveolar rhabdomyosarcoma is difficult to distinguish from embryonal rhabdomyosarcoma concerning patient survival characteristics, gene expression profiles, and CGH arrays [7]. In fact, our work and theirs were not totally contradictory, since they only focused on genomic analysis. As suggested for osteosarcoma, miRNA patterns reflect the phenotypic tumour properties rather than its genetic and could be a promising alternative for RMS diagnosis to surpass the current limitations of molecular analysis combined to traditional histopathology. 

Thus, it seems that miRNA profiling could be very useful for osteosarcoma and rhabdomyosarcoma diagnosis. Here, we showed that on the basis of ten miRNAs, we were able to separate the different subtypes of RMS. We have previously suggested that a panel of five miRNAs was statistically sufficient to distinguish the potent response of osteosarcoma patients to treatment [25]. The TLDA technology presents numerous advantages including its need for few amount of total RNA and the possible analysis of FFPE samples, as it was previously shown by others [47–49]. This method is especially useful to detect circulating miRNAs in patient serum, an emerging field these two past years [50, 51]. A blood-based molecular diagnosis tool through miRNA profiling from patient serum could be a major advance for osteosarcoma, requiring a biopsy for its diagnosis, which could result in a secondary amputation. 

Altogether, these promising results open up the way to a new diagnosis tool based on miRNA for osteosarcoma as well as rhabdomyosarcoma, which could improve patient survival in both cases through the prediction of patient response to chemotherapy and the precise identification of RMS subtypes, respectively.

## Abbreviations

CDDP: Cisplatin
Chondro: Chondrosarcoma cells
Ct: Threshold cycle
Dox: Doxorubicin
FFPE: Formalin-fixed paraffin embedded
ifos: Ifosfamide
KEGG: Kyoto encyclopedia of genes and genomes
LNA: Locked nucleic acid
NT: Non treated
PCA: Principal component analysis
RAD: RAD-001
RMS: Rhabdomyosarcoma
RQ: Relative quantity
RT-qPCR: Real-time quantitative PCR
TLDA: Taqman low density array
15-47: Human osteosarcoma cells CRL-15-47.
